# Piezoelectric Yield of Single Electrospun Poly(acrylonitrile) Ultrafine Fibers Studied by Piezoresponse Force Microscopy and Numerical Simulations

**DOI:** 10.3390/polym16101305

**Published:** 2024-05-07

**Authors:** Margherita Montorsi, Lorenzo Zavagna, Lorenzo Scarpelli, Bahareh Azimi, Simone Capaccioli, Serena Danti, Massimiliano Labardi

**Affiliations:** 1 CNR-IPCF, Pisa Unit, Largo Pontecorvo 3, 56127 Pisa, Italy; margheritamontorsi@cnr.it (M.M.); l.scarpelli1@studenti.unipi.it (L.S.); simone.capaccioli@unipi.it (S.C.); serena.danti@unipi.it (S.D.); 2 PEGASO Doctoral School in Life Sciences, University of Siena, Via Banchi di Sotto 55, 53100 Siena, Italy; l.zavagna@student.unisi.it; 3 Department of Civil and Industrial Engineering (DICI), University of Pisa, Largo Lazzarino 1, 56122 Pisa, Italy; 4 Department of Molecular Medical Surgical Pathology and Critical Area, University of Pisa, Via Savi 10, 56126 Pisa, Italy; bahareh.azimi@ing.unipi.it; 5 CISUP, Center for Instrumentation Sharing of the University of Pisa, Lungarno Pacinotti 43/44, 56126 Pisa, Italy; 6 Physics Department, University of Pisa, Largo Pontecorvo 3, 56127 Pisa, Italy

**Keywords:** poly(acrylonitrile), poly(vinylidene fluoride), piezoelectricity, piezoresponse force microscopy, electrospinning

## Abstract

Quantitative converse piezoelectric coefficient (*d*_33_) mapping of polymer ultrafine fibers of poly(acrylonitrile) (PAN), as well as of poly(vinylidene fluoride) (PVDF) as a reference material, obtained by rotating electrospinning, was carried out by piezoresponse force microscopy in the constant-excitation frequency-modulation mode (CE-FM-PFM). PFM mapping of single fibers reveals their piezoelectric activity and provides information on its distribution along the fiber length. Uniform behavior is typically observed on a length scale of a few micrometers. In some cases, variations with sinusoidal dependence along the fiber are reported, compatibly with a possible twisting around the fiber axis. The observed features of the piezoelectric yield have motivated numerical simulations of the surface displacement in a piezoelectric ultrafine fiber concerned by the electric field generated by biasing of the PFM probe. Uniform alignment of the piezoelectric axis along the fiber would comply with the uniform but strongly variable values observed, and sinusoidal variations were occasionally found on the fibers laying on the conductive substrate. Furthermore, in the latter case, numerical simulations show that the piezoelectric tensor’s shear terms should be carefully considered in estimations since they may provide a remarkably different contribution to the overall deformation profile.

## 1. Introduction

**Figure 1:**
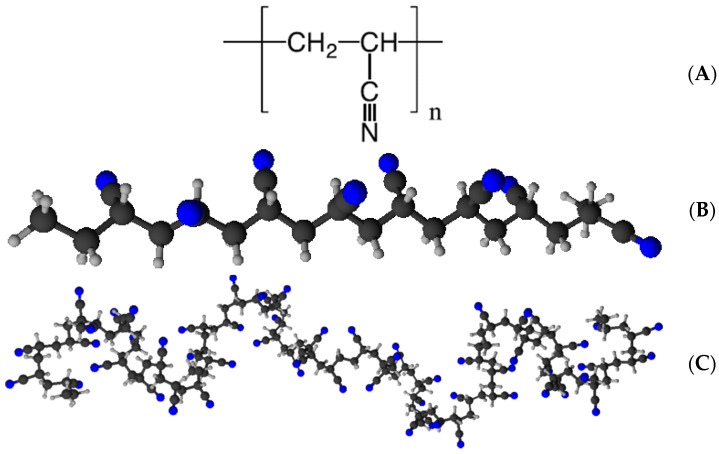
(**A**) Molecular structure of PAN, (**B**) examples of illustrations of its possible syndiotactic zigzag structure and (**C**) of its possible isotactic 3^1^-helical structure [6]. (**B**,**C**) were produced by ChemSketch software (freeware version 2023 1.2).

Polymer-derived piezoelectric ultrafine and nanofibers have become possible alternatives to rigid, brittle ceramic-based piezoelectric materials owing to their high flexibility and biocompatibility [1,2]. Applications include energy scavenging and storage, pressure sensing, biomedicine, tissue engineering, drug release, biosensing, and piezocatalysis [3]. The gold standards in piezoelectric polymers are poly(vinylidene fluoride) (PVDF) and its copolymers. PVDF is a semi-crystalline polymer that exhibits robust piezoelectricity in its β-phase crystalline structure, with a 2.1 debye dipole moment [4]. In recent years, poly(acrylonitrile) (PAN), also named poly(vinyl cyanide), has attracted significant attention for its piezoelectric properties, especially in the form of electrospun fiber meshes. PAN is a vinyl polymer with each repeat unit containing a cyano (-C≡N) group, as shown in Figure 1A. Such a group exhibits a significant dipole moment of 3.9 debye [5]. This work will focus on the PAN polymer, whereas PVDF will be a reference material.

In its secondary structure, PAN tends to assume a 3^1^-helical conformation [7], similarly to poly(l-lactic acid) (PLLA) and to the α-helical one in polypeptides [8]. A less stable planar zigzag conformation, which may look like the β-strand structure in polypeptides [8], could be obtained by straightening of the chains, induced, for instance, by mechanical stretching [9]. Molecular modeling of PAN showed the possibility of realizing the zigzag conformation similar to that in poly(vinyl chloride) [10]. Realistically, the zigzag conformation should exhibit the highest piezoelectric response.

Severe mechanical stretching could promote effective chain alignment and formation of the zigzag secondary structure. If stretching is performed at elevated temperatures or in the presence of the solvent, the formation of elongated and mutually aligned structures, like the zigzag ones, could be allowed by a sufficiently high chain mobility. In case of successive thermal quenching or rapid solvent evaporation, such structures could be maintained in time because of increased viscosity and consequent structural immobilization. Furthermore, mutual chain alignment could also promote polymer crystallization because of the higher degree of order that helps with crystallization seeding. Graphical examples of possible PAN secondary structures are shown in Figure 1B,C.

The tertiary structure of PAN is described as paracrystalline [11], with a pseudo-hexagonal arrangement characterized by poor crystalline ordering, forming a heterogeneous system of amorphous and paracrystalline regions [12]. It is accepted, however, that piezoelectricity in PAN is not strictly related to the existence of a specific crystal phase, as in the case of PVDF. On the contrary, this polymer, as well as other piezopolymers such as PLLA [13], behaves as an electroactive material in which mechanical strain may affect the orientation of its polar moieties by modifying, to some extent, its electrical polarization. To enable this effect, though, a certain degree of coherence among the polar moieties should be present, which could be induced during the solid material formation stage. When crystallization or other self-assembling processes are ineffective, resorting to post-processing procedures, like electrical poling or mechanical stretching, is necessary. Both these actions tend to establish a preferential direction of orientation of the polymer chains. A stable piezoelectric performance can be obtained if such an induced order remains after processing.

In the electrospinning deposition technique, pioneered by J. F. Cooley in 1902 and later refined by Anton Formhals in 1934, a polymeric solution is exposed to a high electric field, thus inducing stretching of the solution into a jet, with the formation of a whipping region of flying fibers, and eventually leading to a dry fibrous mesh collected on the counter-electrode target [14,15,16]. The electrospinning process effectively maximizes the β-phase electroactive fraction in PVDF, being able to realize both piezoelectricity and ferroelectricity [17,18,19]. This method has also been reported to enhance the piezoelectric properties in PAN [20] by severe stretching of the material during the stage of solvent evaporation, hence promoting the formation of aligned molecular configurations. The piezoelectric properties of PAN are correlated to the content of the electroactive phase, namely, the fraction of material in its zigzag conformation. Fourier transform infrared (FTIR) spectroscopy is commonly used to evaluate its content. The vibration band at 1250 cm^−1^ can be assigned to the zigzag conformation of PAN, while the vibration band at 1230 cm^−1^ corresponds to its 3^1^-helical conformation [21,22]. Therefore, the electroactive fraction *Φ* is usually estimated as from Equation (1):

(1) $$\Phi =\frac{S_{1250}}{S_{1250}+S_{1230}}$$

where *S*_1230_ and *S*_1250_ are the peak areas at 1230 cm^−1^ and 1250 cm^−1^, respectively. Literature findings on the piezoelectric yield of PAN specimens and their electroactive phase content are summarized in Table 1, along with the results of our characterizations, which are anticipated here for comparison and will be discussed afterwards.

Other structural features could contribute to the piezoelectric performance of electrospun PAN, such as the degree of alignment of polymer chains induced by the electric fields and electrostatic forces acting during the electrospinning process. Additionally, in rotating electrospinning like the one used in this work to obtain aligned fiber meshes, other factors could influence the formation process of the fibers. Among others, the evaporation rate of the solvent, leading to the formation of the dry fiber, could be enhanced by the airflow induced by the collector’s fast rotation. These aspects are currently under investigation.

The *d*_ji_ piezoelectric coefficient relates the strain *S*_i_ of the material to the internal electric field *E*_j_. In the Voigt notation, the constitutive piezoelectric equation for strain reads [25]

(2) $$S_{i}={c_{ik}^{E}T_{k}+d}_{ji}E_{j}$$

with *T*_k_ being the mechanical stress along direction k, *c*_ik_^E^ the 6 × 6 elastic compliance matrix at constant electric field, and *d*_ji_ the 3 × 6 piezoelectric coupling matrix.

Assessing the electromechanical properties of micro-nanostructured materials on a local length scale is essential for biomedical applications, where interaction with cells happens on the submicrometric scale. For this purpose, detection methods can be applied based on scanning probe microscopies, such as the atomic force microscope (AFM). The piezoelectric functionality can be measured by detecting the surface deformations due to the converse piezoelectric effect after applying an electric potential to an AFM conductive probe (Piezoresponse Force Microscopy, PFM). Recently, an alternative operation mode, named constant-excitation frequency-modulation (CE-FM)-PFM was introduced [26,27], showing the ability to obtain quantitative results for the local *d*_33_ effective piezoelectric coefficient of compliant materials and nanostructures, even when loosely adhered to the substrate. This technique is, therefore, suitable to characterize the electromechanical behavior of single polymeric ultrafine fibers laid on a flat conductive substrate.

This study assesses the local piezoresponse occurring on single ultrafine fibers using CE-FM-PFM. Understanding piezoelectric properties on a submicrometric-to-nanometric length scale could improve the performance of these nanostructures when applied in the bio domain.

## 2. Materials and Methods

### 2.1. Electrospinning of PAN and PVDF Ultrafine Fibers

Poly(acrylonitrile) in the form of powder (average M_w_ of 150 kDa and density of 1.184 g/cm^3^) was acquired from BIOSYNTH (Staad, Switzerland). The chemical structure of this polymer is reported in Figure 1A. Dimethylformamide (DMF) was supplied by Sigma-Aldrich (Milan, Italy). Materials were used without further treatment. Electrospinning was performed using the horizontal setup of a bench apparatus by Linari Engineering s.r.l. (Pisa, Italy). The instrumentation comprises a positive high voltage generator (up to 40 kV), a syringe pump, a glass syringe equipped with a G21 stainless steel blunt-tip needle, and a rotating drum collector (diameter = 8 cm, length = 12 cm). The positive high voltage was connected to the needle and the ground terminal to the collector. PAN was dissolved into DMF at 150 mg/mL concentration to prepare the polymer solution by overnight magnetic stirring at room temperature and 300–400 rpm. The solution was then poured into the glass syringe and electrospun, adopting previously reported parameters [2,20,24] with few adjustments. The needle tip-collector distance was fixed at 15 cm and the flow rate at 0.5 mL/h. The voltage was fixed at 12 kV, and different rotating speeds were tested to achieve random and aligned fiber meshes (5, 1500, 3000 rpm). Electrospinning was performed for 30 min. Finally, PVDF electrospun ultrafine fibers were the same used in previous experiments, obtained as reported earlier [26].

### 2.2. Morphological Characterization

The fibers’ morphological analysis was performed by field emission electron scanning microscopy (SEM) with a Phenom Pro™ Desktop SEM by Thermo Fisher Scientific (Milan, Italy). Before observation, samples were sputter-coated with gold in an S150B Sputter Coater by Edwards High Vacuum International (West Sussex, UK). ImageJ software (version 1.54f) measured the fiber diameters and alignment. A hundred distinct fibers from two mesh pieces were measured to calculate mean diameters and distributions (*n* = 100).

### 2.3. Infrared Spectroscopy

FTIR spectra were recorded with a Nicolet iS20 spectrophotometer (Thermo Fisher Scientific, Waltham, MA, USA) in attenuated total reflectance (ATR) mode for the IR analysis. Spectra were collected using Omnic software (Thermo Fisher Scientific). A resolution of 0.48 cm^−^^1^ was maintained for all measurements, covering the spectral range from 4000 to 525 cm^−1^. The acquisition of spectra was initially performed by averaging 16 scans to check the consistency of the signal. This was followed by an increase to 160 scans to enhance the signal-to-noise ratio. Eventually, all measurements were performed with 320 scans to optimize measurement accuracy.

### 2.4. Piezoresponse Characterization

In this work, piezoresponse force microscopy was implemented by using non-contact type AFM cantilevers (MikroMasch HQ-DPER-XSC11 type C or D, platinum-iridium coated silicon tip, spring constant ~40 N/m, resonant frequency *f*_0_~150–300 kHz, quality factor *Q*_0_~500 in air, tip radius 30 nm), operated in CE-FM-AFM mode [28], with a free cantilever oscillation amplitude of *A*_0_~20 nm. Distance stabilization is obtained by feedback on the oscillation amplitude *A*. An oscillating voltage *V*(*t*) = *V*_dc_ + *V*_ac_ cos(*Ωt*) (*Ω*/2π~80 Hz) is applied to the probe as customary in PFM, and fibers deposited on a conductive, flat substrate, which is connected to the ground, were scanned. The oscillation amplitude signal *A*(*t*) from the FM-AFM controller (PLLProII, RHK Technology, Troy, MI, USA) is demodulated at frequency *Ω* by a dual lock-in amplifier (SRS830DSP, Stanford Research Systems, Sunnyvale, CA, USA), whose amplitude output (∆*A***_Ω_**) was acquired through the auxiliary acquisition channels of our AFM (NanoScope IIIa with MultiMode head, equipped with ADC5 extension and gas cell, Veeco Instruments Inc., Sunnyvale, CA, USA) to form the piezoresponse maps. CE-FM-PFM has already been applied to investigate PVDF-based ultrafine fibers from an electrospun mesh developed for biomedical applications [29]. Here, the same method is also applied to PAN electrospun fibers. For PFM characterization, electrospun fibers were mechanically transferred by gentle contact onto a silicon wafer or a gold-coated glass substrate connected to the ground. The piezoelectric coefficients are obtained by selecting the top region of the fibers within the PFM image and calculating the corresponding data distribution’s average value and standard deviation. The calibration of oscillation amplitude, necessary for determining the *d*_33_ by CE-FM-PFM, was repeated for each fiber. PFM values were normalized by the applied voltage *V*_ac_, typically in the range of 1–2 V_RMS_.

Preliminary measurements of the *d*_31_ piezoelectric coefficient of the produced meshes were obtained using a piezo gauge setup specially developed to address this sample type, as described elsewhere [1]. A stripe of the fiber mesh is clamped between a rigid support and a flexible steel cantilever, acting as a sensitive force gauge. An electric field is applied orthogonally to the mesh thickness by biasing two parallel metal plates. The cantilever bending induced by the piezoelectric stress of the mesh is detected by an optical lever method. Higher measurement sensitivity is obtained by applying an *AC* electric field at the steel cantilever resonant frequency, typically around 150 Hz, by obtaining an amplification effect of 20–30 times.

### 2.5. COMSOL Simulations

**Figure 2:**
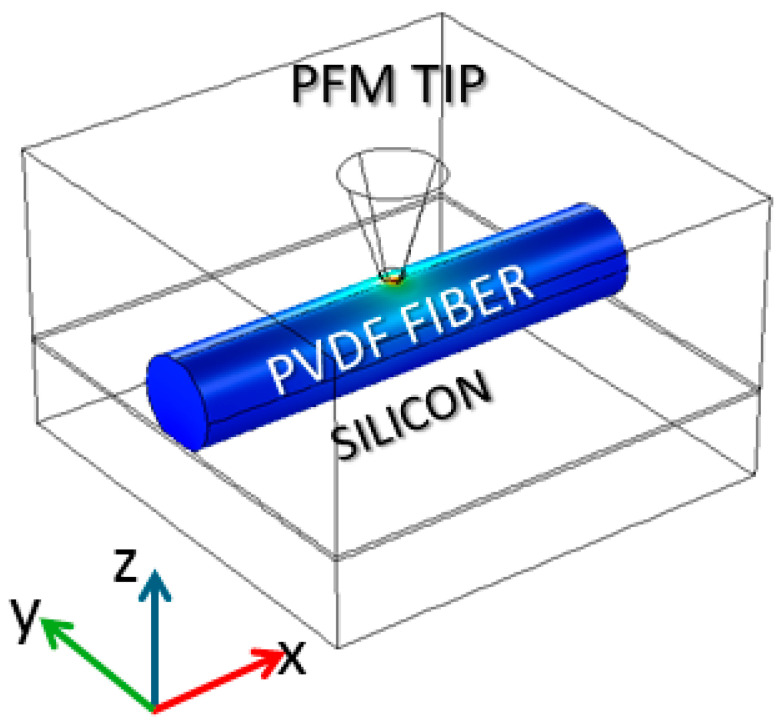
Geometry adopted for the performed numerical simulations, as described in the text.

To gain insight into the piezoresponse behavior of single fibers when detected by PFM, numerical simulations of the system at hand were performed through the COMSOL^TM^ Multiphysics platform. The simulation geometry adopted is reported in Figure 2.

A cylindrical fiber (in two cases: 200 nm and 500 nm diameter) lies on a conductive (silicon) substrate and is oriented along the *X*-axis. The PFM tip is modeled by a cone ending with a hemispherical cap, aligned with the Z axis, facing the fiber’s upper surface. The tip is located very close to the fiber surface, at 20 pm, which is the distance regime pertinent to PFM operated in the employed dynamic mode of AFM. The tip and substrate are set at constant potentials (i.e., 1 V and 0 V, respectively). The Z displacement of the fiber surface at the tip apex location is assumed to be the amount of piezoresponse of the sample.

The piezoelectric coupling matrix for PVDF, available from the COMSOL library, was used at first for calculations. Its representation in Voigt notation is the following:

(3) $$d_{ij}=\left(\begin{array}{cccccc} 0 & 0 & 0 & 0 & 0 & 0\\ 0 & 0 & 0 & 0 & 0 & 0\\ 13.6 & 1.5 & -33.8 & 0 & 0 & 0 \end{array}\right)pm/V$$

This pristine matrix describes poled PVDF, with its polar axis oriented along Z. We remark, however, that PVDF should be described more appropriately by a coupling matrix including the shear coefficients *d*_15_ and *d*_24_. An example of such a matrix, which we have also adopted for further simulations, is as reported in [30]:

(4) $$d_{ij}=\left(\begin{array}{cccccc} 0 & 0 & 0 & 0 & -27 & 0\\ 0 & 0 & 0 & -23 & 0 & 0\\ 21 & 1.5 & -32.5 & 0 & 0 & 0 \end{array}\right)pm/V$$

Rotation of the reference system by an angle β around the X axis was applied in COMSOL only to the fiber to simulate the change of polar axis orientation: parallel to the tip axis for β = 0° and perpendicular to the tip axis for β = 90°. In this way, the mechanical compliance and piezoelectric coupling matrices should be rotated accordingly, thus providing a realistic description of the material’s properties.

## 3. Results

### 3.1. Characterization of PAN Fiber Meshes

**Figure 3:**
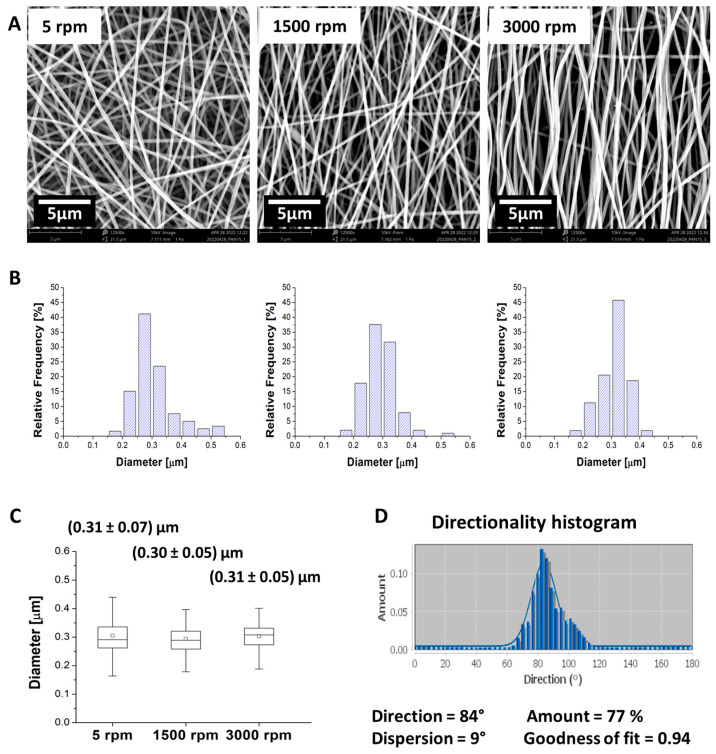
(**A**) SEM micrographs of electrospun PAN meshes collected at different rotation speeds. (**B**) Related diameter distributions. (**C**) Box plots of the fiber diameter distributions obtained at different rotation speeds and associated mean and standard deviations. (**D**) Directionality histogram and mesh parameters collected at 3000 rpm, obtained with the FFT directionality analysis function of ImageJ software.

The solution used for fibrous mesh production appeared homogeneous and transparent in color. As analyzed via ImageJ software, the samples yielded a mean fiber diameter of 0.29–0.30 µm for all the rotation speeds tested. Representative SEM micrographs of PAN fibers collected at different rotation speeds are reported in Figure 3. The Taylor cone, jet, and whipping regions were visible and stable during electrospinning. Overall, 30 min electrospinning time led to the formation of handleable but highly electrostatic meshes, the latter feature being associated with charge retention [31]. Increasing the rotation speed resulted in higher spatial dispersion of the fibers, attributed to the air movement generated by the rotating drum. This has led to the production of broader and thinner meshes as far as the rotation speed was increased, transitioning from a thickness of 20 ± 5 µm for randomly oriented fiber meshes collected at 5 rpm to 9 ± 3 µm for uniaxially aligned fiber meshes collected at 3000 rpm. The consistency of the diameters obtained at all the tested rotating speeds suggests that the 3000 rpm speed was appropriate to produce the desired alignment without exerting a further stretching effect on the fibers.

ATR-FTIR of the pristine PAN powder and the three fiber meshes was conducted to derive the fraction of the electroactive zigzag phase. As expected, the powder only shows the 1230 cm^−^^1^ characteristic band related to the 3^1^ helical conformation, formed spontaneously due to its higher thermodynamic stability. The fiber samples obtained by three different speeds of the rotating collector show the compresence of the 1250 cm^−^^1^ band related to the electroactive zigzag structure instead. The randomly aligned fibers, obtained at the 5 rpm rotation speed, exhibit a zigzag fraction of 24%, while the more aligned meshes have higher fractions, namely 46% for the fibers obtained at 1500 rpm and 33% for those obtained at 3000 rpm.

**Figure 4:**
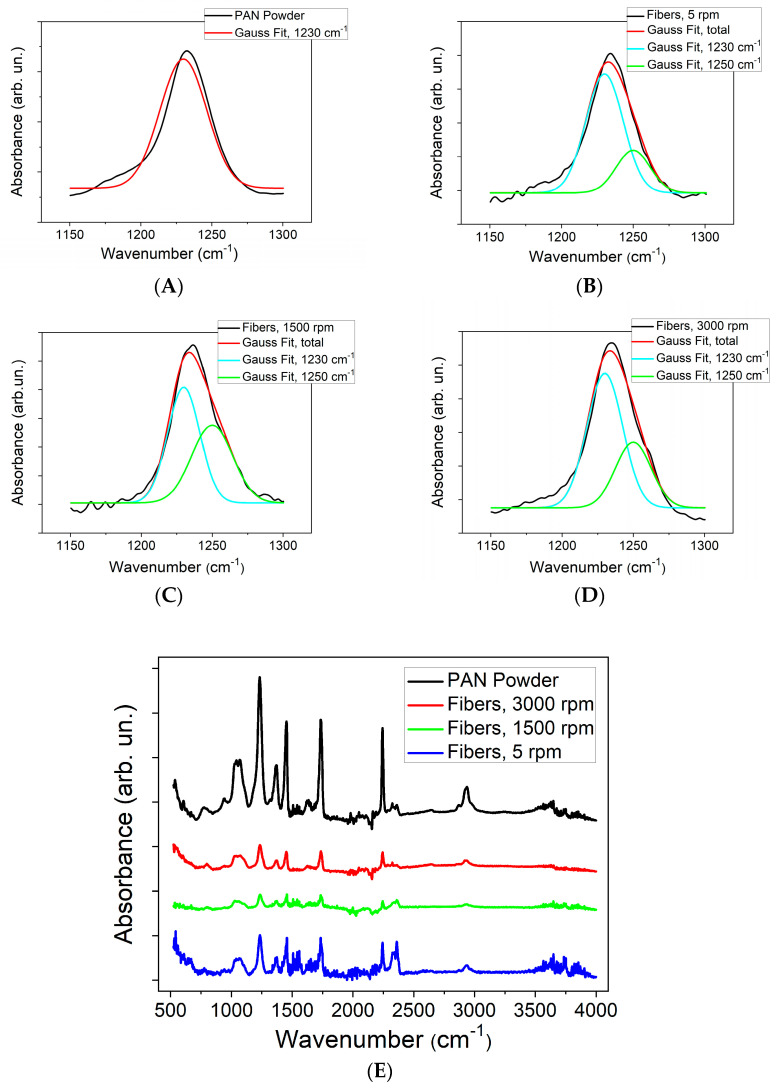
ATR-FTIR spectra of (**A**) pristine PAN powder; (**B**–**D**) PAN ultrafine fiber samples electrospun at different collector velocities: (**B**) 5 rpm, (**C**) 1500 rpm, (**D**) 3000 rpm; and (**E**) wide-range IR spectra for all samples (vertical axes were shifted for better graphical representation).

Figure 4 reports all FTIR spectra of our electrospun fiber samples as well as of the used PAN powder. Fits to determine the electroactive fractions according to Equation (1) were performed by setting the wavenumber of the two absorption bands to 1230 cm^−1^ and 1250 cm^−1^ as fixed while letting both Gaussian peak width and area be free fitting parameters. Fit results for such bands are shown in Figure 4A–D, while the broad range spectra are reported in Figure 4E.

The transverse piezoelectric yield of electrospun PAN fiber meshes can be studied by a piezo gauge apparatus [1], which can derive the effective *d*_31_ coefficient resulting from the transverse piezoelectric effect of the whole fiber structure. Preliminary results provided the transverse piezoelectric performance of random fiber meshes (*d*_31_ = 20 ± 16 pm/V) [32], whereas the positive control on a commercial, uniaxially stretched, and poled PVDF film (from Goodfellow Cambridge Ltd., Huntingdon, UK) provided a value of 45 ± 6 pm/V, and a negative control on a PEOT/PBT random electrospun mesh provided 2.1 ± 1.7 pm/V. More thorough characterization and analysis of these meshes are not the focus of the present work and will be reported in forthcoming publications. Nonetheless, the relation between the piezoelectric performance of macroscopic devices and the ones of the constituent materials and nanostructures is indeed an intriguing issue deserving more intense research efforts.

**Figure 5:**
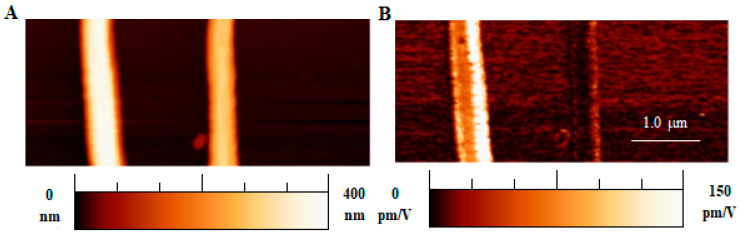
(**A**) Topography, (**B**) corresponding effective *d*_33_ map of two PAN fibers from the same mesh (obtained at 3000 rpm collector speed).

Figure 5 shows the AFM topography and the corresponding CE-FM-PFM scan of two PAN fibers. The fibers exhibit a smooth, non-porous morphology as visible from all topographic maps in this study and from SEM micrographs in Figure 3A. Although the two fibers of Figure 5 belong to the same mesh, a marked difference in their piezoresponse is recorded.

**Figure 6:**
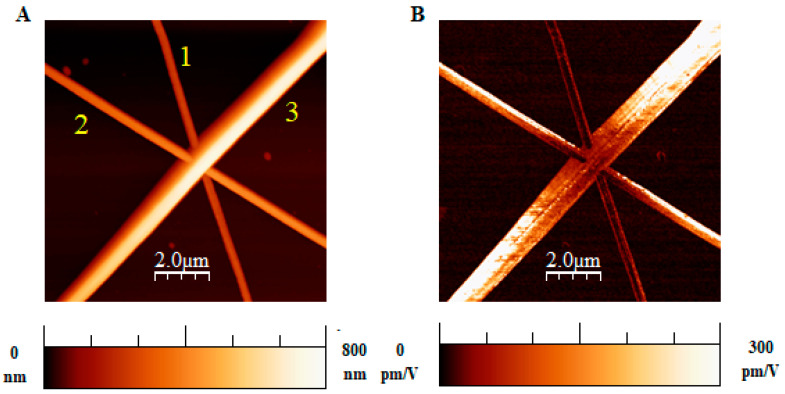
(**A**) Topography and (**B**) corresponding effective *d*_33_ map of three overlapped fibers of different kinds: (1) PAN @ 3000 rpm, (2) PAN @ 5 rpm, and (3) PVDF fiber used as a reference. PFM signals are lower in the overlapping region, where fibers are farther away from the conductive substrate.

Figure 6 shows PFM results for three fibers of different sizes. In this case, the crossing between a PVDF fiber and two PAN fibers is displayed, where the three kinds of fibers are overlaid on the substrate by transferring them successively along different angles. The observed decrease of piezoresponse at the crossing location stems from the more considerable distance from the substrate of the overlapping fibers.

**Figure 7:**
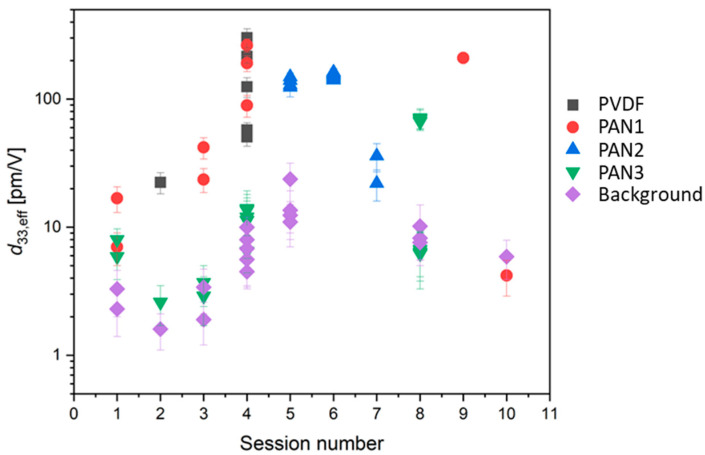
Effective piezoelectric coefficient *d*_33,eff_ of several fibers of different types: PAN @ 5 rpm (PAN1), PAN @ 1500 rpm (PAN2), PAN @ 3000 rpm (PAN3), PVDF fibers used as a reference (PVDF), as compared to background signal (Background), indicative of typical measurement noise. Data for session #4 correspond to the image of Figure 6, while those for session #8 correspond to the image of Figure 5.

Various piezoresponse values were recorded on different fibers, as summarized in Figure 7. This illustrates the ability of CE-FM-PFM to characterize the local piezoresponse of compliant polymeric nanostructures, like the present ultrafine fibers, or even polymeric nanoparticles [33], loosely adhered to the substrate, conveniently and reliably.

The measurements were conducted over two months on fibers transferred on different substrates (e.g., silicon and gold) and with different AFM probes. The typical acquisition time for PFM scans reported in this work was about 1–3 h per image. In Figure 7, each plotted point is associated with one acquired image.

As evident from the reported data, a wide range of values was obtained for the piezoelectric yield of different fiber types and fibers of the same kind. The range found for the fibers deposited at the 5 rpm collector speed was from 4 to 210 pm/V, with an average of 88 pm/V; the one for fibers obtained at 1500 rpm was from 29 to 146 pm/V, with an average of 88 pm/V, and the one for fibers obtained at 3000 rpm was from 3 to 70 pm/V, with an average of 17 pm/V, as also summarized in Table 1. These values refer to a small number of measurements and, therefore, are affected by high statistical error. Furthermore, a more refined statistical analysis of the present data collection is impeded for the following reasons. The wide range of values obtained could lead to the conclusion that the piezoelectric performance of single fibers is highly variable, probably due to the complex formation process of electrospinning. Still, it could also be due to the measurement mechanism of PFM, where the detected piezo displacement depends on the direction of the polar axis of the fiber when transferred onto the conductive substrate. Therefore, the measured values reflect not only the inherent piezoelectric performance of our fibers but also the preparation method of the samples for PFM analysis.

A general finding was a smaller piezoresponse for PAN fibers with a diameter of less than 300 nm, irrespective of the drum rotation speed. Furthermore, piezoelectric coefficient values even higher than the typical values for the bulk materials were interestingly evidenced for some of the fibers, both for PAN and PVDF.

Figure S1 of the Supplementary Materials reports all PFM images corresponding to the data of Figure 7. Figure S2 of the Supplementary Materials reports line profiles along some of the fibers, showing the trend of detected piezoresponse as a function of the position along the fiber in the case of overlapping fibers.

**Figure 8:**
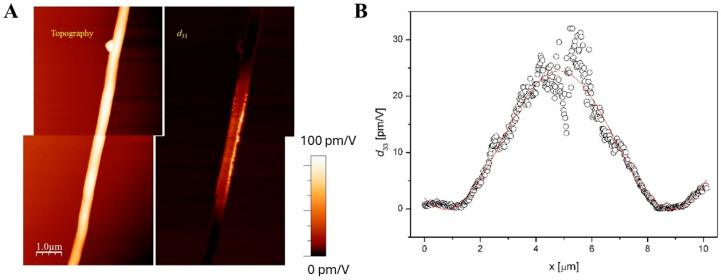
(**A**) Topography (left) and corresponding effective *d*_33_ map (right) of an electrospun PVDF fiber deposited on gold. (**B**) Profile of *d*_33_ along the fiber length (symbols) and sinusoidal fit (solid line).

Piezoresponse variation along the fiber axis was also observed in some instances. A clear example with a PVDF fiber, similar to the ones characterized in [29] and used here as a reference, is reported in Figure 8, in which a gradual change of the piezoresponse signal is visible. Repeated scans of the same area (not shown) confirmed that the polarization structure was stable during typical scanning periods (e.g., a few hours). A sinusoidal fit of the *d*_33_ profile value along the fiber length is reported in Figure 8B, showing good agreement and supporting the assumption of an increasingly twisted polarization vector direction along the fiber. This could be because the polarization axis of the polymer was twisted, either during the electrospinning process or during transfer to the conductive substrate. Another possible case of axis twisting for a PAN fiber can be seen in Figure S1 (part D, AC) of the Supplementary Materials.

### 3.2. Results of COMSOL Simulations

To rationalize the high variability of PFM results and the presence of cases with a gradual change of piezoresponse along the fiber, numerical simulations were exploited by resorting to the COMSOL^TM^ Multiphysics platform.

**Figure 9:**
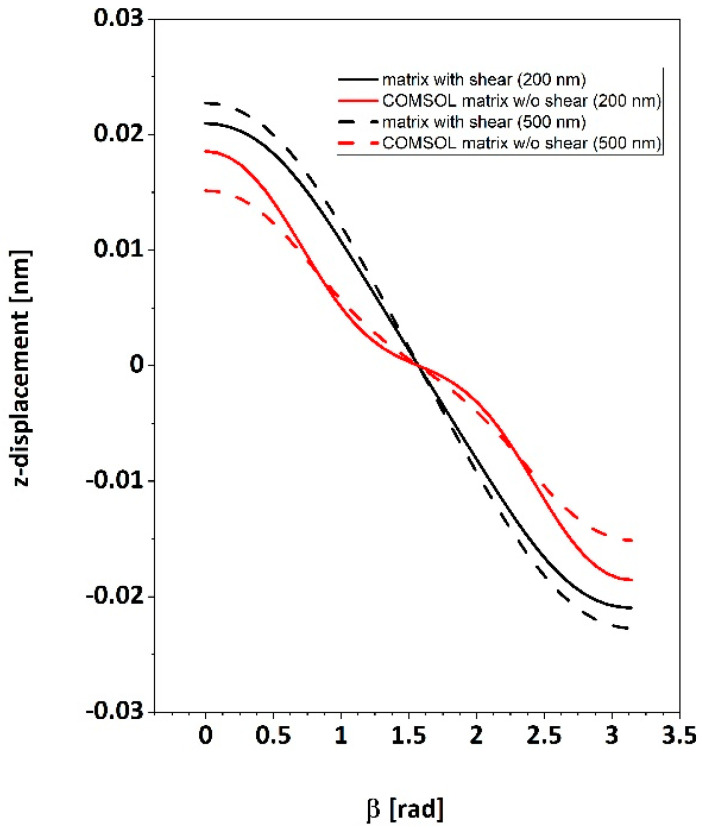
Simulated piezoresponse as a function of the polar axis direction, β, for two different fiber diameters and two different forms of the piezoelectric coupling matrix (see text).

Piezoresponse results for the two different fiber diameters (i.e., 200 nm and 500 nm) as a function of polar axis orientation β, are shown in Figure 9. Curves in red color show the trend of piezoresponse by using the built-in COMSOL piezoelectric coupling matrix (Equation (3)) on the full range of β angles. It is found that the signal profile does not follow a regular sinusoidal trend as could be expected. Still, an additional undulation is visible, with three oblique inflection points at β around 45°, 90°, and around 135°. By adopting instead the piezoelectric coupling matrix incorporating shear terms (Equation (4)), the expected sinusoidal trend of the signal as a function of the rotation angle β is recovered.

**Figure 10:**
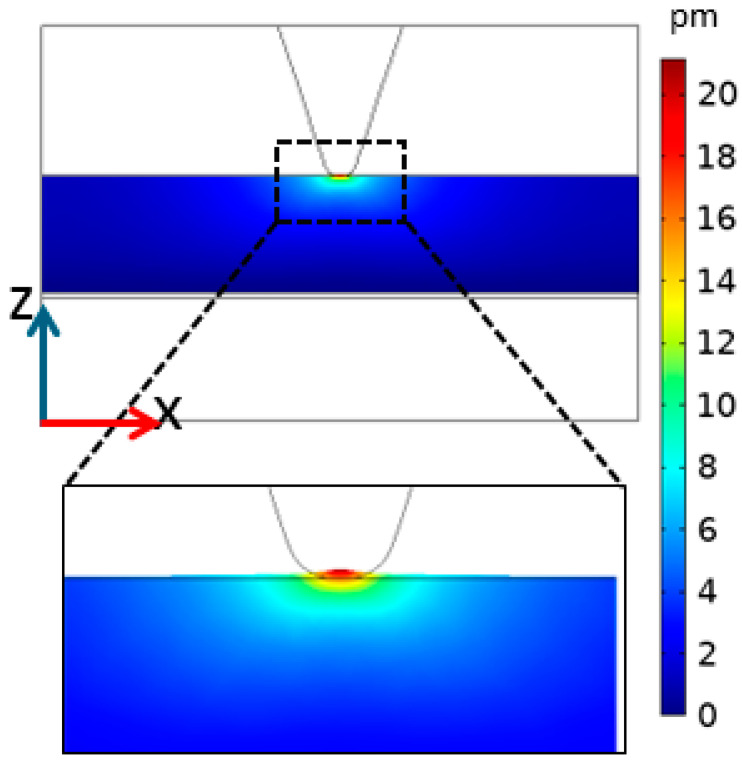
Simulated Z-displacement field due to piezoelectric effect. In the zoomed inset at the bottom, displacement has been amplified by a factor of 200 in the Z direction for illustration purposes.

Figure 10 shows a detailed displacement field in the Z direction obtained from our simulations after applying a potential difference of 1 V. Although the deformed region of the sample lies entirely beneath the probe apex, a larger sample volume is involved in such deformation, where both translational and shear movements may contribute to the overall effect. A detailed investigation of this aspect is beyond the scope of this work.

## 4. Discussion

First, it should be noted that PAN is the first material in electrospun ultrafine fibers, after PVDF, to exhibit piezoelectricity as detected through the CE-FM-PFM method. Exploration of local piezoelectric properties provides different information with respect to macroscopic measurements of the same property. Whereas the global behavior could represent the final aim of research developments, especially for application to sensors and actuators, knowledge of the sample structure and functionality down to the nanometer scale helps to elucidate the reasons for the observed behaviors and to improve the strategy to obtain the desired results. Additionally, local behavior could be more relevant than global behavior in cases where the devised micro/nanostructure should be used as a support or host for different substances, gas or chemical sensors, or biological scaffolds. For instance, piezoelectric fibers and/or particles could present a random orientation of their polar axes so that the macroscopic efficiency of the system used as a sensor or actuator may result in being too weak; however, when a guest material fills the spaces between the fibers, a new interface is created, which could enable the desired functional behavior. In case the direct piezoelectric effect stimulates cell growth or differentiation [3], only the local effect near cell receptors should be relevant despite the global effect averaged over the scaffold.

In this work, we investigated the piezoelectric behavior of PAN ultrafine fibers on a submicrometric scale. We used a long acquisition time for two main reasons:

(i) The *AC* electric field frequency Ω cannot exceed around 100 Hz to allow the correct operation of the AFM frequency-modulation mode [26]; this limits the sampling time (time spent on each measurement pixel of the map) to around 30 ms. A map composed of 256 × 256 pixels takes around one hour to be acquired, as each scan line must be run twice, forward and backward;
(ii) For the correct quantitative operation of the CE-FM mode of PFM, the distance stabilization feedback loop cannot be too fast [26]. Therefore, the scanning speed should be reduced to avoid possible damage or the picking up of organic material by the probe scanning over the fibers. A ruined tip would compromise the quality of the following images, forcing probe replacement to retrieve the proper performance of the microscope. To preserve the probe, we have experienced that the scanning speed should not exceed around 100 nm/s.

A damaged tip usually results in a substantial and unrealistic increase in the PFM signal; some of the highest piezoresponse values reported could be due to this unwanted condition. Of course, an inspection of nanostructure piezoresponse does not necessarily need to scan an entire image. Still, it could be enough to position the probe on the top of the desired nanostructure and record the corresponding signal. However, imaging is always preferable since it allows us to check the correct performance of the microscope and to spot changes in the scan behavior that could indicate occasional damage or material pickup of the probe.

The obtained results from numerous measurement sessions on the various ultrafine fiber meshes available can be classified as follows:

(i) Uniform piezoresponse is typically detected along the length of single fibers, as in Figure 5. The variability of the reported *d*_33_ effective values could be ascribed to the different polar axis directions of fibers lying on the conductive substrate;
(ii) Gradual signal variations at fiber crossing sites are observed. This can be explained by the probe’s greater distance from the substrate and the related decrease in the electric field inside the fibers;
(iii) Occasionally, the piezoresponse of a single fiber may vary even when fully resting on the substrate. This result could be explained by assuming the gradual twisting of the fiber around its longitudinal axis, which may lead to a corresponding rotation of the polar axis of the fiber’s material.

As already mentioned, standard analysis of variance (ANOVA) statistics would not provide meaningful results for our data collection since our measured values are influenced not only by the inherent piezoelectric performance of single fibers but also by the way such fibers were deposited on the measurement substrate, specifically by the direction of their polar axis, which is a further independent variable of a random character. Average values provide a significant indication of piezo performance; however, to obtain a more meaningful comparison among single fibers, a method should be devised to deposit all fibers with the coherent orientation of their polar axes. Work in this direction is currently in progress.

Numerical simulations performed through the COMSOL^TM^ Multiphysics platform can also be exploited to support the above assumptions. Indeed, simulations with larger fiber diameters led to lower piezoresponse values of about 20% between 200 nm and 500 nm. Using the coupling matrix which included shear coefficients instead, the sinusoidal trend observed experimentally was reproduced, and the signal variation between the different diameters was reduced to about 10%. Such a more negligible difference is expected since the piezoresponse should be ruled essentially by the volume of material nearest to the probe and not by the farther regions, so the total piezo displacement should be almost independent of the sample thickness. Therefore, it can be concluded that the matrix incorporating shear coefficients provides a more realistic description of the resulting piezoresponse effect.

## 5. Conclusions

We applied CE-FM-PFM to single electrospun PAN ultrafine fibers laid onto a conductive flat substrate to investigate piezoelectric properties on a submicrometric scale. We found a remarkable piezoelectric response of the fibers with a diameter larger than 300–400 nm, while the thinner fibers (i.e., diameter < 300 nm) typically showed much weaker piezoelectric activity. In some cases, fibers can show a variable local piezoresponse along their length, compatible with a possible gradual twisting of their polar axis. In other cases, uniform piezoresponse was recorded along the fiber, at least on a length scale of several microns. Both cases comply with the hypothesis of a uniform polar direction perpendicular to the fiber axis. Numerical simulations, performed to check to what extent the observed behavior was to be expected, revealed that the piezoelectric coupling matrix should necessarily incorporate the shear terms *d*_15_ and *d*_14_ to provide the experimentally observed trends of piezoresponse as a function of polar axis orientation. If such terms were not included, the piezoresponse profile would not exhibit the observed sinusoidal trend but rather a more complicated pattern with an intermediate change in slope.

In conclusion, nanoscale exploration of the local functional properties of nanostructured materials appears to be a critical factor in rationalizing the performances of devices and scaffolds for biosensing, regenerative medicine, and other biomedical applications.

## Figures and Tables

**Table 1:** Piezoelectric constants measured for different PAN samples and the zigzag (electroactive) phase fraction determined via FTIR spectroscopy according to Equation (1). Note: 10^−8^ cgs esu correspond to 0.333 pC/N = 0.333 pm/V; DMF: dimethylformamide; DMSO: dimethylsulfoxide.

Material	Sample Type/Treatment	Measuring Method	Piezoelectric Constant/ Voltage Output	Zigzag Content [%]	Reference
Solvent cast films, poled and stretched. PAN M_w_ = 35 kDa. Solvent: DMF.	Poled (5 × 10^4^ V/cm) Unstretched	Dynamic stress and piezoelectric polarization (strain of 1.5 × 10^−3^, oscillating frequency of 11 Hz) used to obtain absolute values of *d*_31_.	*d*_31_ = 0.32 × 10^−8^ cgs esu	Not reported	[23]
	Poled (5 × 10^4^ V/cm) Stretched		*d*_31_ = 1.7 × 10^−8^ cgs esu		
	Poled (10 × 10^4^ V/cm) Stretched		*d*_31_ = 3 × 10^−8^ cgs esu		
Electrospun fiber meshes with different degrees of alignment. PAN M_w_ = 90 kDa. Solvent: DMF.	With/without charge removal	Periodic pressure applied. Voltage/current output of the mesh reported.	Voltage outputs of 0.2–1.2 V/cm^2^ upon compression	80–86%	[20]
Electrospun fiber meshes with different diameter distributions. PAN M_w_ = 150 kDa. Solvent: DMF.	No annealing	Piezoresponse force microscopy (PFM). *d*_33_ of the fibers reported.	*d*_33_ = 2–14 pm/V	55–75%	[2]
	60 °C annealing		*d*_33_ = 1–16 pm/V	45–80%	
	95 °C annealing		*d*_33_ = 8–40 pm/V	70–90%	
Electrospun fiber meshes, PAN with various tacticites. Solvent: DMSO.	Commercial (M_w_ = 300 kDa)	Periodic semi-static normal load method. Effective *d*_33_ of the mesh reported.	*d*_33,eff_ = 0–0.2 pC/N	Not reported	[24]
	25% Isotactic (M_w_ = 857 kDa)		*d*_33,eff_ = 1.5–2.0 pC/N		
	52% Isotactic (M_w_ = 517 kDa)		*d*_33,eff_ = 0.4–0.6 pC/N		
Electrospun fiber composite meshes of PAN/BaTiO_3_.	0% w BaTiO_3_	Periodic pressure applied. Voltage/current output of the mesh reported.	0.64 V/cm^2^	45%	[21]
	5% w BaTiO_3_		0.94 V/cm^2^	50%	
	10% w BaTiO_3_		1.36 V/cm^2^	52%	
	15% w BaTiO_3_		1.86 V/cm^2^	56%	
	20% w BaTiO_3_		1.56 V/cm^2^	53%	
	25% w BaTiO_3_		1.26 V/cm^2^	51%	
Electrospun fiber meshes collected at different rotation speeds. PAN M_w_ = 150 kDa. Solvent: DMF.	5 rpm (random)	Piezoresponse force microscopy (PFM). *d*_33_ of the fibers reported.	*d*_33_ = 88 ± 44 pm/V	24%	This work
	1500 rpm		*d*_33_ = 88 ± 58 pm/V	46%	
	3000 rpm (aligned)		*d*_33_ = 17 ± 11 pm/V	33%	

## Data Availability

The data presented in this study are available in the present article and the related Supplementary Materials.

## Supplementary Materials

The following supporting information can be downloaded at https://www.mdpi.com/article/10.3390/polym16101305/s1, Figure S1 (parts A, B, C, D): PFM maps of different PAN and PVDF fibers, Figure S2: line profiles of PFM signals along some of the fibers in Figure S1.
