# Supplementary material for: Piezoelectric Yield of Single Electrospun Poly(acrylonitrile) Ultrafine Fibers Studied by Piezoresponse Force Microscopy and Numerical Simulations
Source: Polymers (Basel). 2024 May 7;16(10):1305. doi: 10.3390/polym16101305 (PMC11125114; doi:10.3390/polym16101305)
Supplement: Supplementary file 1 [file polymers-16-01305-s001.zip › polymers-2973949-supplementary.pdf]

polymers

Supplementary

Piezoelectric Yield of Single Electrospun Poly(acrylonitrile) Ultrafine Fibers Studied by Piezoresponse Force Microscopy and Numerical Simulations

Margherita Montorsi [1], Lorenzo Zavagna [2], Lorenzo Scarpelli [1,3], Bahareh Azimi [4], Simone Capaccioli [1,5,6], Serena Danti [1,3,5] and Massimiliano Labardi [1,5,*]

1. CNR-IPCF, Pisa Unit, Largo Pontecorvo 3, 56127 Pisa, Italy; margheritamontorsi@cnr.it (M.M.); l.scarpelli1@studenti.unipi.it (L.S.); simone.capaccioli@unipi.it (S.C.); serena.danti@unipi.it (S.D.)
2. PEGASO Doctoral School in Life Sciences, University of Siena, Via Banchi di Sotto 55, 53100 Siena, Italy; l.zavagna@student.unisi.it
3. Department of Civil and Industrial Engineering (DICI), University of Pisa, Largo Lazzarino 1, 56122 Pisa, Italy
4. Department of Molecular Medical Surgical Pathology and Critical Area, University of Pisa, Via Savi 10, 56126 Pisa, Italy; bahareh.azimi@ing.unipi.it
5. CISUP, Center for Instrumentation Sharing of the University of Pisa, Lungarno Pacinotti 43/44, 56126 Pisa, Italy
6. Physics Department, University of Pisa, Largo Pontecorvo 3, 56127 Pisa, Italy

* Correspondence: massimiliano.labardi@cnr.it; Tel.: +39-050-2214322

Citation: Montorsi, M.; Zavagna, L.; Scarpelli, L.; Azimi, B.; Capaccioli, S.; Danti, S.; Labardi, M. Piezoelectric Yield of Single Electrospun Poly(acrylonitrile) Ultrafine Fibers Studied by Piezoresponse Force Microscopy and Numerical Simulations. *Polymers* **2024**, *16*, 1305. https://doi.org/10.3390/polym16101305

Academic Editor: Yinsong Si

Received: 4 April 2024
Revised: 30 April 2024
Accepted: 3 May 2024
Published: 7 May 2024

Copyright: © 2024 by the authors. Licensee MDPI, Basel, Switzerland. This article is an open access article distributed under the terms and conditions of the Creative Commons Attribution (CC BY) license (https://creativecommons.org/licenses/by/4.0/).

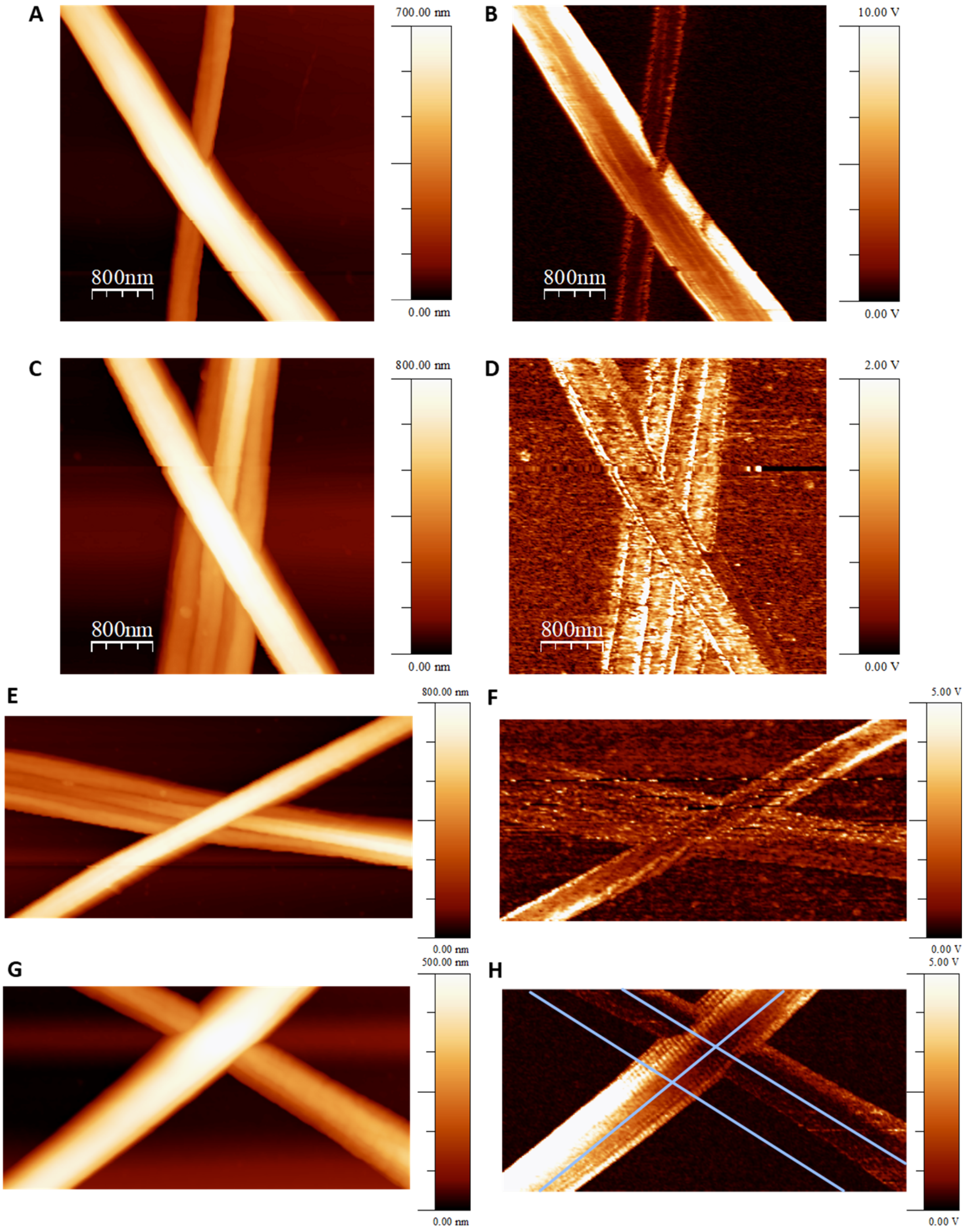

Figure S1. (part A). PFM maps of different PAN and PVDF electrospun fibers. (A) Topography: PVDF fiber, diameter 600 nm; PAN3 fiber, diameter 280 nm; (B) PFM, Scale 1 V = 34.4 pm/V (PVDF), 30.1 pm/V (PAN). (C) Topography: PAN1 fiber, diameter 650 nm; PAN3 (3 fibers), diameter 300-400 nm; (D) PFM, Scale 1 V = 6.6 pm/V. (E) Topography: PAN1 fiber, diameter 650 nm; PAN3 (3 fibers), diameter 300-400 nm; (F) PFM, Scale 1 V = 6.6 pm/V. (G) Topography: PVDF fiber, diameter 400 nm; PAN1 fiber, diameter 250 nm; (H) PFM, Scale 1 V = 11 pm/V.

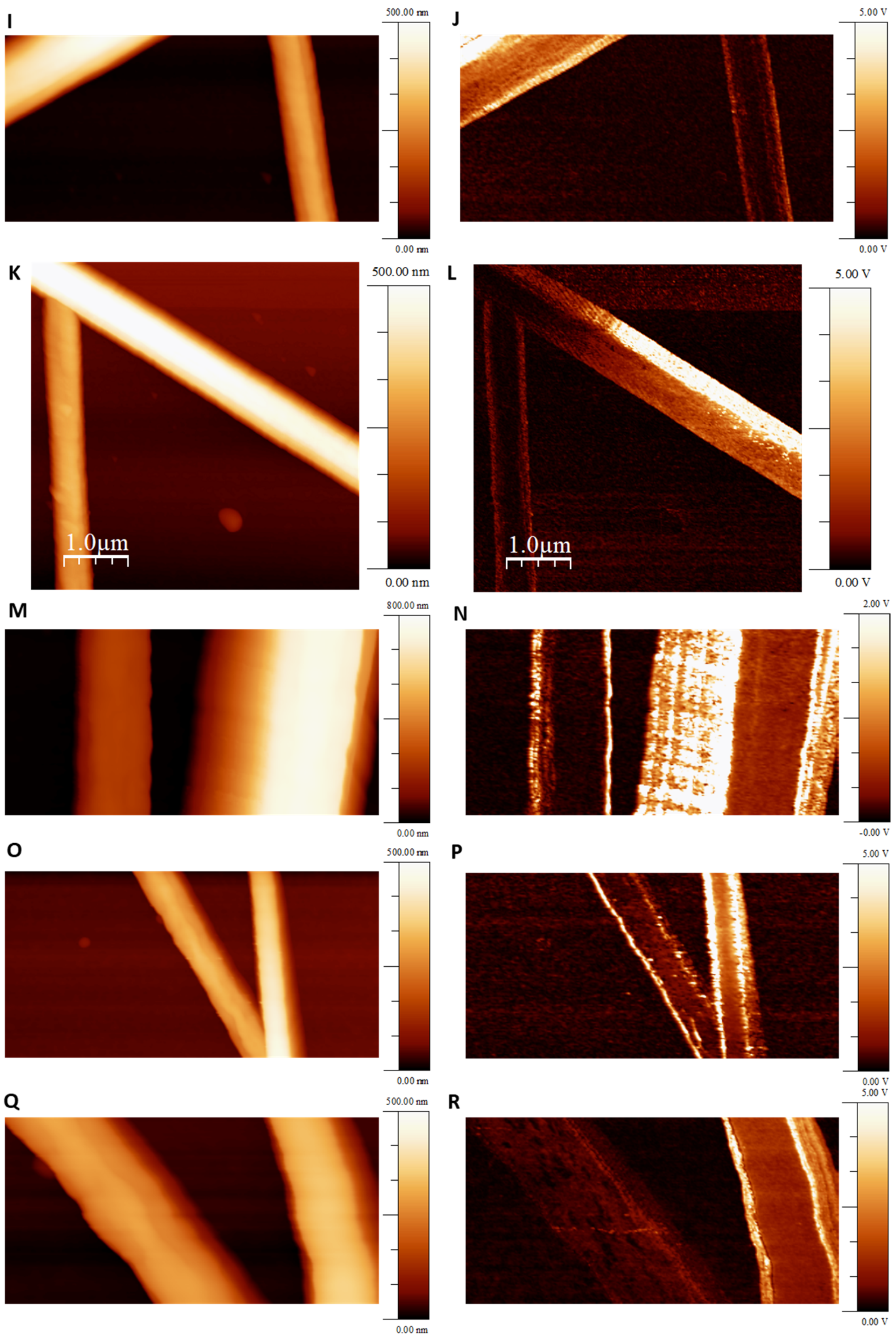

Figure S1. (part B). (I) Topography: PVDF fiber, diameter 400 nm; PAN3 fiber, diameter 300 nm; (J) PFM, Scale 1 V = 11 pm/V. (K) Topography: PAN1 fiber, diameter 450 nm; PAN3 fiber, diameter 300 nm; (L) PFM, Scale 1 V = 11 pm/V. (M) Topography: PVDF fiber, diameter 750 nm; PAN3 fiber, diameter 250 nm; (N) PFM, Scale 1 V = 113 pm/V. (O) Topography: PAN2 fiber, diameter 280 nm; PAN2 fiber, diameter 380 nm; (P) PFM, Scale 1V = 28.6 pm/V. (Q) Topography: PAN2 fiber, diameter 280 nm; PAN2 fiber, diameter 380 nm; PFM, Scale 1 V = 113 pm/V.

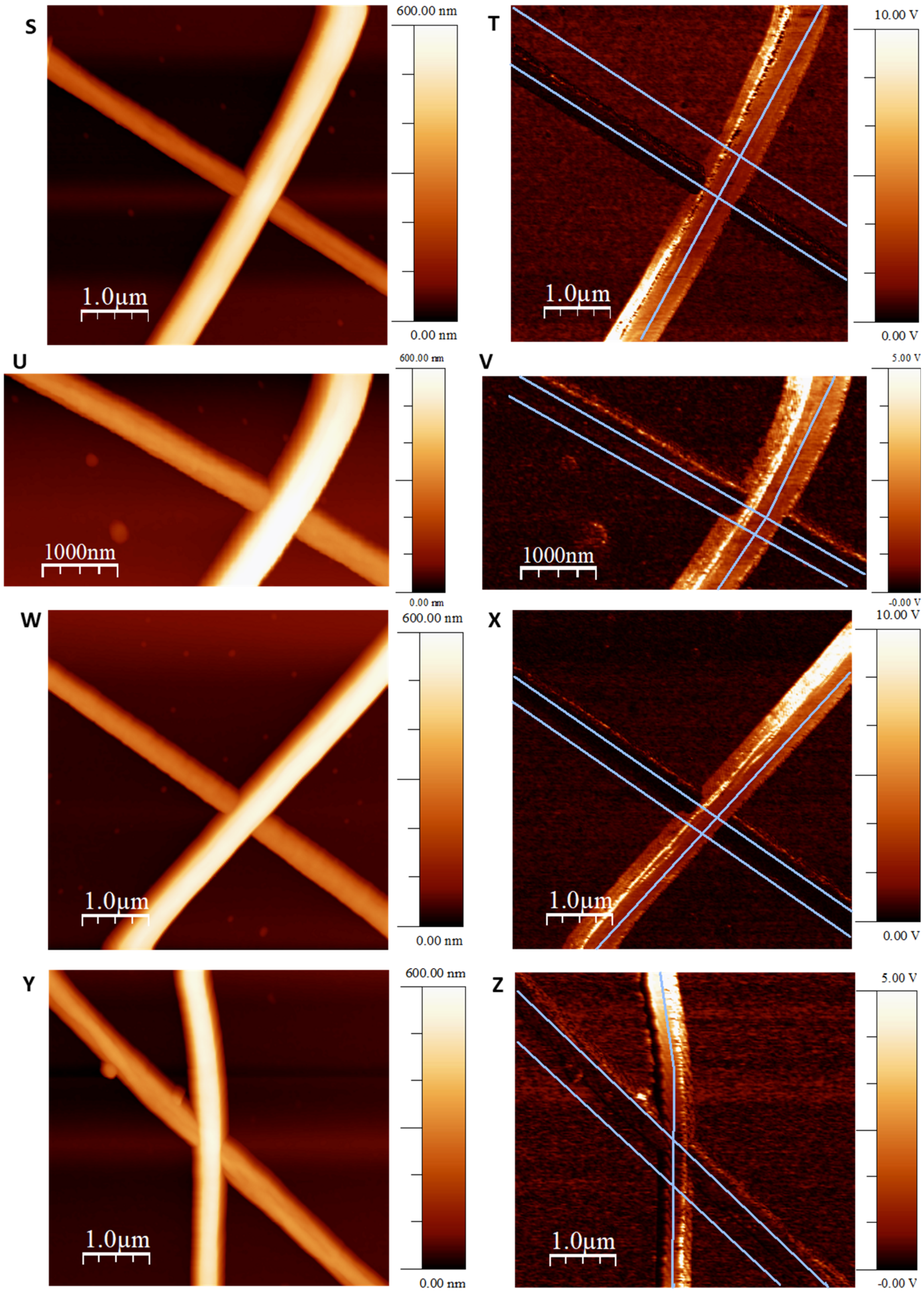

Figure S1. (part C). (S) Topography: PVDF fiber, diameter 500 nm; PAN1 fiber, diameter 230 nm; (T) PFM, Scale 1 V = 29.3 pm/V. (U) Topography: PVDF fiber, diameter 550 nm; PAN1 fiber, diameter 270 nm; (V) PFM, Scale 1 V = 29.3 pm/V. (W) Topography: PVDF fiber, diameter 500 nm; PAN1 fiber, diameter 250 nm; (X) PFM, Scale 1 V = 29.3 pm/V. (Y) Topography: PAN1 fiber, diameter 500nm; PAN1 fiber, diameter 300 nm; (Z) PFM, Scale 1 V = 29.3 pm/V.

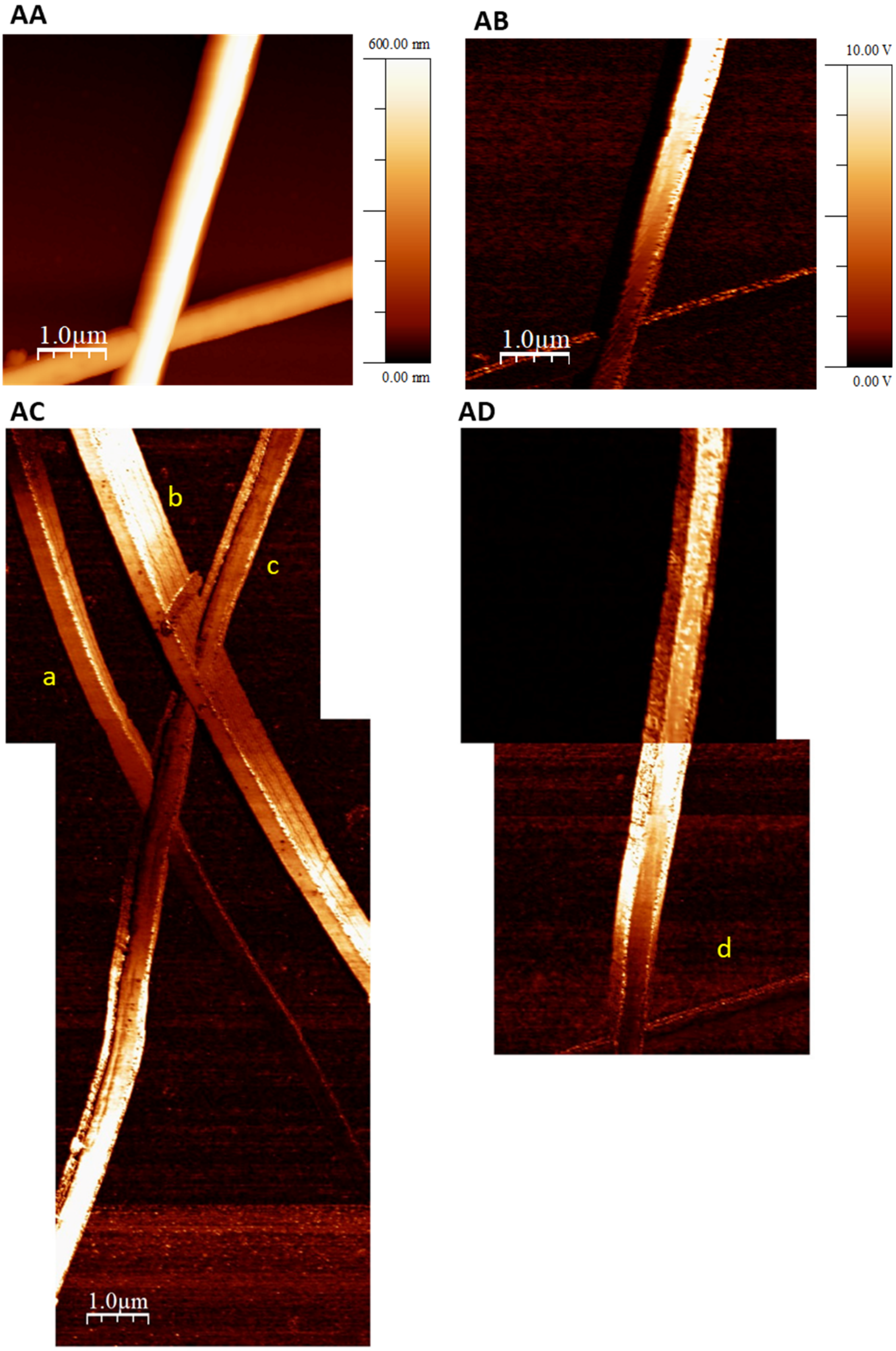

Figure S1 (part D). (AA) Topography: PAN1 fiber, diameter 550 nm; PAN1 fiber, diameter 320 nm; (AB) PFM, Scale 1 V = 22 pm/V. (AC-AD) Stitched PFM images: (AC) Topography: PAN1 (a) fiber, diameter 460 nm; PAN1 (b) fiber, diameter 680 nm; PAN1 (c) fiber, diameter 550 nm; (AD) Topography: PAN1 (d) fiber, diameter 320 nm.

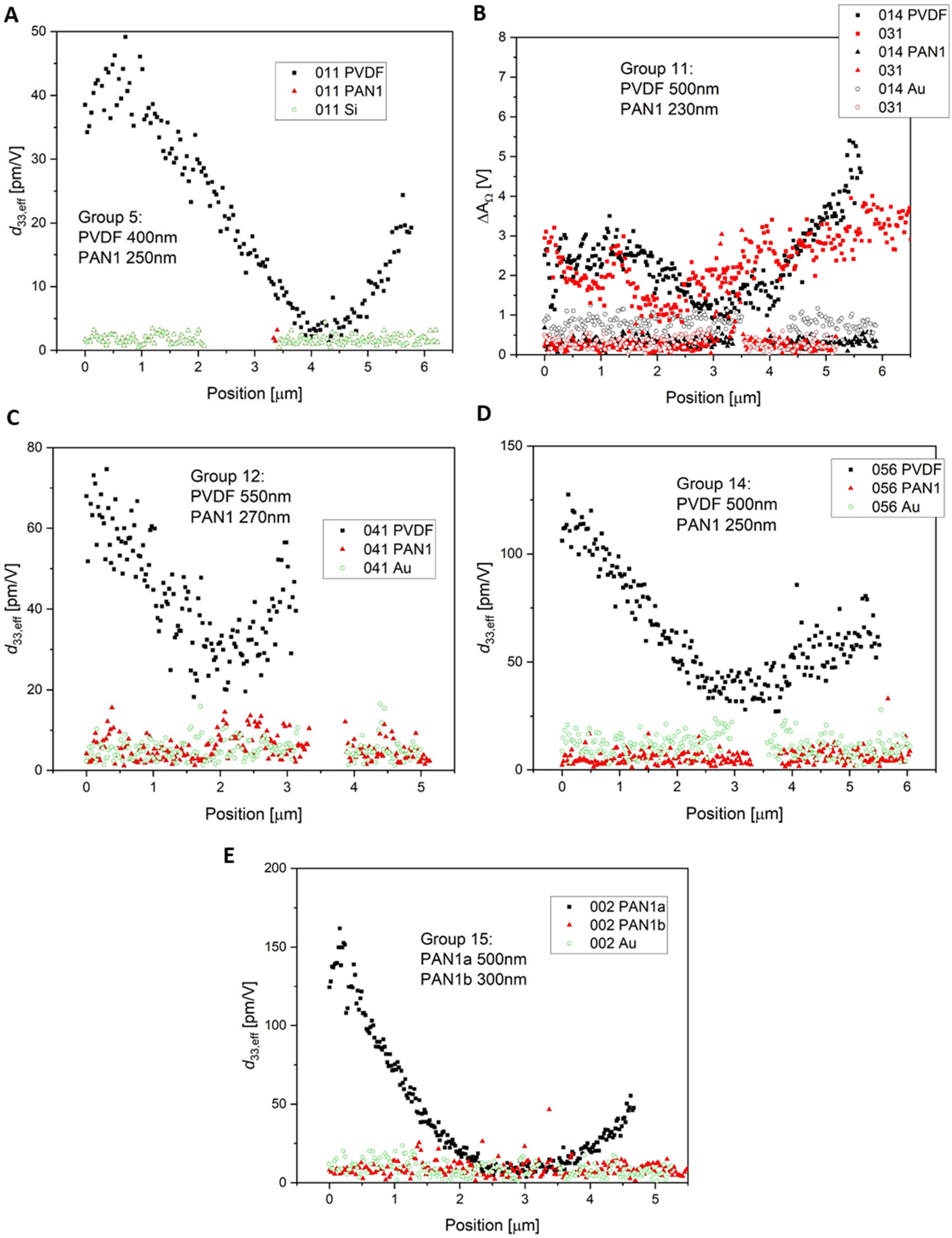

Figure S2. Line profiles of PFM signals along some of the fibers in Figure S1, namely: (A) Figure S1 (part A, H); (B) Figure S1 (part C, T), PFM, Scale 1 V = 29.3 pm/V; (C) Figure S1 (part C, V); (D) Figure S1 (part C, X); (E) Figure S1 (part C, Z). .
